# Infectious Complications Following Snakebite by *Bothrops lanceolatus* in Martinique: A Case Series

**DOI:** 10.4269/ajtmh.19-0369

**Published:** 2019-10-14

**Authors:** Dabor Resiere, Hossein Mehdaoui, Rémi Névière, Claude Olive, Mathieu Severyns, Adeline Beaudoin, Jonathan Florentin, Yannick Brouste, Rishika Banydeen, André Cabié, Bruno Mégarbane, José María Gutiérrez, Hatem Kallel

**Affiliations:** 1 Department of Critical Care, University Hospital of Martinique, Fort-de-France, France;; 2 Department of Cardiology, University Hospital of Martinique, Fort-de-France, France;; 3 Department of Microbiology, University Hospital of Martinique, Fort-de-France, France;; 4 Department of Orthopedic Surgery, University Hospital of Martinique, Fort-de-France, France;; 5 Department of Infectious Diseases, University Hospital of Martinique, INSERM CIC 1424, Antilles University, Fort-de-France, France;; 6 Department of Medical and Toxicological Critical Care, Lariboisière Hospital, Paris-Diderot University, INSERM UMRS1144, Paris, France;; 7 Instituto Clodomiro Picado, Facultad de Microbiología, Universidad de Costa Rica, San José, Costa Rica;; 8 Intensive Care Unit, Cayenne General Hospital, Cayenne, France

## Abstract

Infections secondary to snakebite occur in a number of patients and are potentially life-threatening. *Bothrops lanceolatus* bites in Martinique average 30 cases per year and may result in severe thrombotic and infectious complications. We aimed to investigate the infectious complications related to *B. lanceolatus* bite. A retrospective single-center observational study over 7 years (2011–2018) was carried out, including all patients admitted to the hospital because of *B. lanceolatus* bite. One hundred seventy snake-bitten patients (121 males and 49 females) were included. Thirty-nine patients (23%) presented grade 3 or 4 envenoming. Twenty patients (12%) developed wound infections. The isolated bacteria were *Aeromonas hydrophila* (3 cases), *Morganella morganii* (two cases), group A *Streptococcus*, and group B *Streptococcus* (one case each). Patients were treated empirically with third-generation cephalosporin (or amoxicillin–clavulanate), aminoglycoside, and metronidazole combinations. Outcome was favorable in all patients. The main factor significantly associated with the occurrence of infection following snakebite was the severity of envenoming (*P* < 0.05). Our findings clearly point toward the frequent onset of infectious complications in *B. lanceolatus*–bitten patients presenting with grade 3 and 4 envenoming. Thus, based on the bacteria identified in the wounds, we suggest that empiric antibiotic therapy including third-generation cephalosporin should be administered to those patients on hospital admission.

## INTRODUCTION

Snakebites account for about 1.8–2.7 million envenomings and 81,000–138,000 deaths per year worldwide.^1^ In Martinique, about 30 cases of snakebite are recorded every year. *Bothrops lanceolatus*, a member of the Viperidae family, Crotalinae subfamily, is the only venomous species encountered in Martinique.^2^ *Bothrops lanceolatus* bite may result in severe thrombotic complications, including cerebral, pulmonary, and myocardial infarction, as well as coagulation disorders and endothelial injuries, which could be fatal or involve long-term sequelae.^2–4^ Thus, envenomed patients should promptly receive a specific antivenom to prevent such severe complications.

Snakebites are frequently responsible for local complications combining pain and local edema in the minutes following the bite, followed, in severe cases, by local necrosis and blistering. Wound infection may contribute to tissue necrosis, bacteremia, and even septic shock.^5,6^ Like in envenomings by other snakes, such infectious complications are routinely observed following *B. lanceolatus* bite, but their precise incidence is unknown.

The oral bacterial flora of *B. lanceolatus* includes *Aeromonas hydrophila*, *Morganella morganii*, *Klebsiella pneumoniae*, *Bacillus* spp., and *Enterococcus* spp.^7^ These bacteria are usually found in post-snakebite abscesses, suggesting that they have been inoculated in the wound from the snake oral cavity, thus supporting the possible need for empiric antibiotic treatment after the snakebite, particularly in cases associated with prominent local tissue damage. Interestingly, local effects of the venom, such as tissue necrosis, edema, and vascular damage, constitute a favorable environment for bacterial growth.

Because data regarding the risk and outcome of infectious complications resulting from *B. lanceolatus* bite are poorly known, we designed this observational study to determine the incidence of wound infection in patients bitten by this species and describe the involved bacteria and the patients’ outcome.

## PATIENTS AND METHODS

We conducted a retrospective single-center observational study at the University Hospital of Martinique from January 1, 2011 to September 4, 2018. In Martinique, all *B. lanceolatus*–bitten patients are referred to our hospital because the BothroFav^®^ antivenom is only available at our Emergency Department.

All patients admitted to the hospital for snakebite by *B. lanceolatus* during the study period were included. Patients with a history of snakebite but without medical or computer records and patients with a history of bite but without evidence of envenoming were excluded. Our database has been registered at the Commission Nationale de l'Informatique et des Libertés (registration n° 2213908 v 0.) in compliance with the French law on electronic data sources.

### Data collection.

Patients were selected using the medical information department database, the antivenom dispensing list, and the emergency department records. Clinical and biological data were collected from the patient medical records and the various emergency department software (Dx Care, X-plore, and cyberlab). We collected the usual demographic, clinical, biological, microbiological, management, and outcome data. The signs suggestive of *B. lanceolatus* bite, the date of bite onset, the bite zone, and the time between the bite and antivenom administration (if administered) were sought. Monthly rainfall and maximal temperatures recorded in Martinique were obtained from the French national meteorological service (Météo France).

### Diagnosis and management of snakebite wound infection.

Wound infection following snakebite was defined as the presence of at least two local suggestive signs or as the presence of fever and/or chills and one local suggestive sign. Fever was defined as body temperature above 38°C measured using tympanic thermometer. Local signs suggestive of wound infection included pain, erythema, local warmth, swelling, lymphangitis, purulence, delayed healing, crepitus in soft tissues, discolored or friable granulation tissue, and wound breakdown or dehiscence, as previously listed.^8,9^ Because our study was retrospective, if no abnormality was mentioned in the patient record, it was assumed that no infectious complication had resulted from the snakebite.

In patients with local signs of infection, samples obtained from blood cultures, local sampling in case of purulence, and wound culture if patients had surgical debridement were sent to the bacteriology laboratory to identify the involved bacteria. Samples were subjected to Gram staining and examined for bacterial growth. They were plated on nonselective blood agar and chocolate agar and cultured at 37°C for 2–7 days, and the color and shape of the colonies were observed. Species identification was performed with API-20E and API-20NE systems (BioMérieux, Marcy L’Etoile, France). Antimicrobial susceptibilities of all isolates were determined by the disk diffusion method based on the definition of the Antibiogram Committee of the French Microbiology Society.^10^ The inhibition zone diameter of each drug for each isolate was determined after overnight incubation at 35.8°C in ambient air. The interpretative criteria of the inhibition zone and minimum inhibitory concentrations were in accordance with those of the Antibiogram Committee of the French Society of Microbiology.^10^ Bacteremia caused by coagulase-negative staphylococci or *Bacteroides* sp. was defined as two positive results of two independent blood cultures of samples obtained from two distinct peripheral veins.

Severity of the snakebite was graded as previously reported (Table 1).^11^ The snakebite was considered as severe if graded 3 or 4. When microbiological cultures were positive for a microorganism from the skin flora (except blood cultures positive for coagulase-negative staphylococci or *Bacteroides* sp.), clinical and laboratory data were analyzed to differentiate true infection from colonization.

**Table 1:** Severity score of envenoming after *Bothrops lanceolatus* bite (adapted from ref. 11)

Grade	Severity	Symptoms
1	Minor	No swelling
		No pain
		No general signs
2	Moderate	Local swelling confined to two segments of the bitten limb
		Moderate pain
		No general signs
3	Severe	Regional edema, extension of swelling beyond two segments of the bitten limb
		Persistent and resistant pain to analgesics
		No general signs
4	Major	Swelling spreading to the trunk
		General signs (vomiting, headache, and abdominal or chest pain)
		Hypotension
		Isolated thrombocytopenia
		Disseminated intravascular coagulation

Severity is defined by at least one confirmed item.

Patients were managed by the physicians in charge according to the usual national and international guidelines. Administration of BothroFav antivenom was decided according to the recommendations.^1,11^ In our hospital, combinations of third-generation cephalosporin (or amoxicillin–clavulanate), aminoglycoside, and metronidazole are routinely prescribed at admission to patients with grade 3–4 envenoming, and during stay, to patients with signs of infection regardless of the degree of envenoming.

### Statistical analysis.

Continuous variables are expressed as mean ± SD. Categorical variables are expressed as number (percentage). Differences between groups were assessed using Student’s *t*-tests for continuous variables and Chi-squared tests for categorical variables. Correlation between variables was determined using linear regression. Data were analyzed using the Excel (2007) and SPSS program version 24. *P*-values < 0.05 were considered as significant.

## RESULTS

During the 8-year study period, 170 patients (age: 45 ± 18 years, including seven children (4%); male-to-female gender ratio of 2.5) were referred to our hospital for snakebite management (Table 2).

**Table 2:** Clinical parameters recorded in 170 *Bothrops lanceolatus*–bitten patients on hospital admission

Variables	Total patients (*N* = 170)	Infected patients (*N* = 20)	Noninfected patients (*N* = 150)	*P*-value
Age (years)	45 ± 18	48 ± 15	45 ± 18	0.4
Male, *N* (%)	121 (71%)	15 (75%)	106 (71%)	0.7
Hospitalization, *N* (%)	107 (63%)	20 (100%)	87 (58%)	< 0.0001
Past medical history				
Snakebite, *N* (%)	10 (6%)	1 (5%)	9 (6%)	0.9
Immunosuppression, *N* (%)	4 (2%)	2 (10%)	2 (1%)	0.02
Cardiovascular risk, *N* (%)	28 (17%)	3 (15%)	25 (17%)	0.9
Coagulopathy, *N* (%)	4 (2%)	2 (10%)	2 (1%)	0.02
Snakebite characteristics				
Time from envenoming to admission (hours)	3.5 ± 4.3	3.7 ± 4.7	3.5 ± 4.3	0.8
Snake captured, *N* (%)	45 (27%)	8 (40%)	37 (25%)	0.1
Site of the bite, *N* (%)				0.8
Upper limb	71 (42%)	10 (50%)	61 (41%)	
Lower limb	98 (58%)	10 (50%)	88 (59%)	
Buttock	1 (1%)	0	1 (1%)	
Local bleeding, *N* (%)	91 (54%)	11 (55%)	80 (53%)	0.9
Local pain, *N* (%)	163 (96%)	19 (95%)	144 (96%)	0.833
Envenoming grade, *N* (%)				
1	22 (13%)	0	22 (15%)	–
2	109 (64%)	8 (40%)	101 (67%)	–
3	33 (19%)	8 (40%)	25 (17%)	–
4	6 (4%)	4 (20%)	2 (1%)	–
Clinical presentation and complications				
Heart rate (beat/min)	80 ± 16	79 ± 16	80 ± 16	0.9
Temperature (°C)	36.8 ± 0.8	37.1 ± 0.7	36.8 ± 0.5	0.7
Systolic arterial pressure (mmHg)	137 ± 24	128 ± 27	139 ± 23	0.04
Diastolic arterial pressure (mmHg)	80 ± 15	75 ± 14	81 ± 15	0.1
Mean arterial pressure (mmHg)	99 ± 16	93 ± 17	100 ± 16	0.05
SpO_2_ (%)	99 ± 2	99 ± 1	98 ± 2	0.07
Shock, *N* (%)	3 (1.8%)	3 (15%)	0	–
Consciousness impairment, *N* (%)	3 (1.8%)	3 (15%)	0	–
Convulsion, *N* (%)	1 (0.6%)	1 (5%)	0	–
Thrombosis, *N* (%)	1 (1%)	0	1 (1%)	–
Compartmental syndrome, *N* (%)	6 (4%)	5 (25%)	1 (1%)	–
Bacteremia, *N* (%)	3 (2%)	3 (15%)	0	–
Laboratory parameters on admission				
Creatine kinase (IU/L)	300 ± 283	311 ± 257	298 ± 287	0.9
Platelet count (G/L)	238 ± 67	213 ± 76	241 ± 65	0.07
Prothrombin index (%)	96 ± 13	92 ± 17	97 ± 12	0.09
Activated partial thromboplastin time (minutes)	31.5 ± 3.7	30.6 ± 3.6	31.6 ± 3.7	0.3
Fibrinogen (g/L)	3.0 ± 0.7	2.9 ± 1.0	3.0 ± 0.6	0.7
C-reactive protein (mg/L)	7 ± 42	31 ± 118	4 ± 7	0.009
White blood cells (G/L)	7.8 ± 2.7	9.3 ± 3.6	7.6 ± 2.4	0.005
Antivenom management				
Antivenom administration, *N* (%)	154 (91%)	19 (95%)	135 (90%)	0.5
Number of vials	1.7 ± 1.3	2.4 ± 1.5	1.6 ± 1.3	0.016
Time from snakebite to antivenom administration (hour)	6.0 ± 7.0	6.5 ± 8.9	5.9 ± 6.8	0.8
Time from admission to antivenom administration (hour)	3.2 ± 5.3	4.3 ± 7.5	3.1 ± 5.0	0.4
Antivenom reinjection, *N* (%)	19 (12%)	10 (53%)	9 (7%)	< 0.001
Empiric antibiotic administration, *N* (%)	37 (22%)	17 (85%)	20 (13%)	–
Amoxicillin–clavulanate, *N* (%)	11 (6%)	2 (10%)	9 (6%)	–
Third-generation cephalosporin, *N* (%)	17 (10%)	6 (30%)	11 (7%)	–
Gentamycin, *N* (%)	12 (7%)	4 (20%)	8 (5%)	–
Metronidazole, *N* (%)	12 (7%)	5 (25%)	7 (5%)	–

### Incidence.

**Figure 1.**
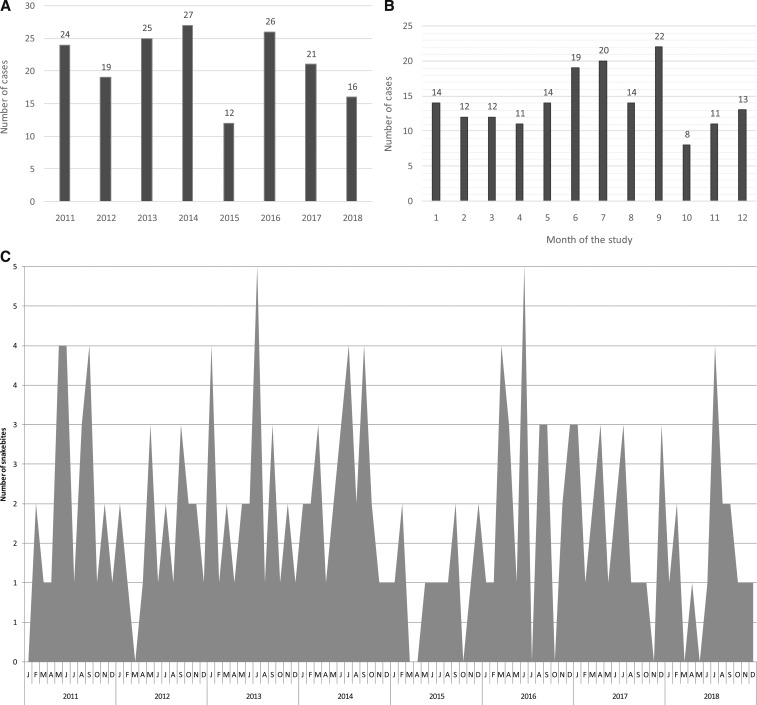
Distribution of the 170 *Bothrops lanceolatus* bite cases according to the year (**A**) and month (**B** and **C**) of the study.

**Figure 2.**
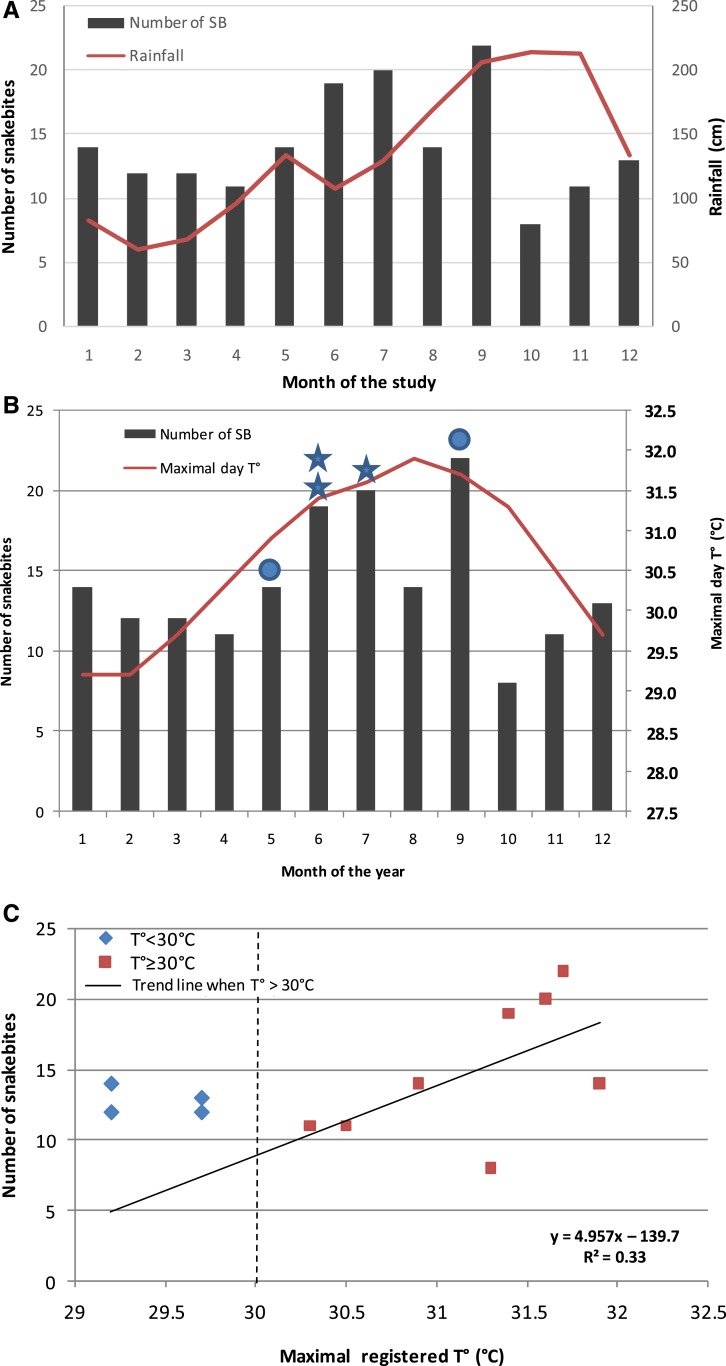
Relationship between the monthly distribution of *Bothrops* snakebites and the recorded rainfall (**A**) and maximal temperatures (**B** and **C**). The line shows the trend in snakebites when the recorded maximal temperature is above 30°C. Stars represent cases with *Aeromonas hydrophila* infection and circles represent cases with *Morganella morganii* infection. This figure appears in color at www.ajtmh.org.

The number of snakebites was 21 cases per year (Figure 1A), corresponding to an incidence rate of six bites per 100,000 inhabitants per year in Martinique. Monthly distribution of snakebites showed peak incidence in June, July, and September, with an average of two bites per month (Figure 1B and C). No significant relationship between the seasonal incidence of snakebite and precipitation registered by the French national meteorological service was observed (Figure 2A), whereas the number of snakebites significantly increased when the recorded maximal temperature was above 30°C (*R*^2^ = 0.33; Figure 2B and C).

### Presentation and post-snakebite infection onset.

**Figure 3.**
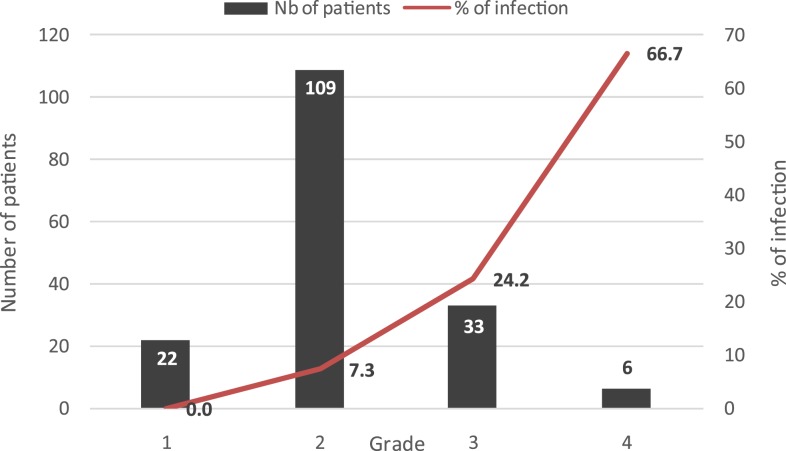
Prevalence of infection according to the grade of envenoming. This figure appears in color at www.ajtmh.org.

On hospital admission, 39 patients (23%) presented with grade 3 or 4 envenoming. Twenty patients (12%) had clinical signs suggestive of post-snakebite infections. Bacteriological samples were positive in seven cases (35%). The isolated bacteria were *M. morganii* in two cases, *A. hydrophila* in three cases, *Streptococcus* A in one case, and *Streptococcus* B in one case. All isolated *M. morganii* and *A. hydrophila* were susceptible to third-generation cephalosporins. The main factor associated with the occurrence of infection following snakebite was the severity of the bite. Twelve patients (31%) developed infection in the severely envenomed patients versus eight (6%) in the non-severely envenomed patients (*P*< 0.0001; Figure 3).

### Management and outcome.

Seventy-nine patients (46%) were admitted to the medical ward, 25 (15%) to the intensive care unit (ICU), and three (2%) to the surgical ward, whereas 63 (37%) were discharged after management in the emergency department. Almost all patients (93%) were treated with the specific BothroFav antivenom. It is noteworthy that patients presenting infections more frequently required antivenom readministration than those without infection (53% versus 7%, *P*< 0.001; Table 2). Based on the severity of the envenoming grade and the suspicion of local infection, 37 patients received one antibiotic or a combination of antibiotics. The following antibiotics were administered empirically: third-generation cephalosporin in 17 (10%) patients, amoxicillin–clavulanate in 11 (6%) patients, gentamycin in 12 (7%) patients, and metronidazole in 12 (7%) patients.

**Figure 4.**
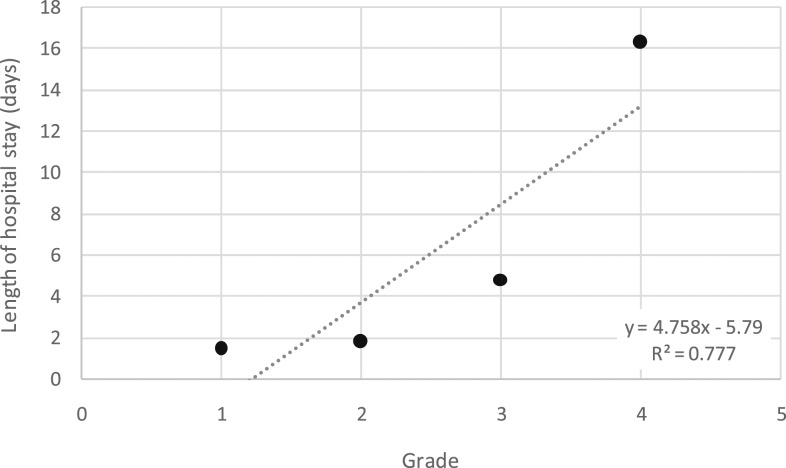
Length of hospital stay according to the grade of envenoming in 170 *Bothrops lanceolatus*–bitten patients.

The complications observed during hospitalization are reported in Table 3. No myocardial infarction or brain stroke occurred. No patient died. Length of hospital stay was 3 ± 5 days (6 ± 9 days in the ICU versus 3 ± 4 days in the other hospital wards, *P* = 0.01). Length of hospital stay significantly increased according to the severity grade of the snakebite (*R*^2^ = 0.77; Figure 4) and was significantly longer in patients with infection (11 ± 10 versus 2 ± 1 days, *P* < 0.0001).

**Table 3:** Local signs recorded in 170 infected and noninfected *Bothrops lanceolatus*–bitten patients

	Total patients (*N* = 170)	Infected patients (*N* = 20)	Noninfected patients (*N* = 150)
Increasing pain	28 (17%)	20 (100%)	8 (5%)
Abscess	7 (4%)	7 (35%)	0
Erythema	17 (10%)	16 (80%)	1 (1%)
Cellulitis	4 (2%)	4 (20%)	0
Necrosis	5 (3%)	5 (25%)	0
Necrotic fasciitis	1 (1%)	1 (5%)	0
Gangrene	0	0	0

## DISCUSSION

Infection following *B. lanceolatus* bite is relatively frequent (12% in our case series), and patients at highest risk are those presenting with severe envenoming (grades 3 and 4). The bacteria responsible for wound infection are those commonly isolated from the snake mouth, suggesting that the main source of contamination comes from the snake causing the bite.

Wound infection following snakebite usually accounts for 9–77% of the bitten patients, as described in several studies (Table 4).^5,6,8,12–15^ The large differences in the reported prevalence of secondary infections in snakebites between different studies can be related to variations in the criteria used to establish the presence of infection. A strict criterion is the laboratory isolation and identification of bacteria from the affected tissues or blood in envenomed patients. However, clinical criteria are also used to diagnose infection. In this regard, discrepancies may arise because some clinical manifestations of local infection can also be caused by the action of venom toxins in the tissue, associated with inflammation. In our study, infection was defined as the presence of two of the following local signs: pain, erythema, local warmth, swelling, lymphangitis, purulence, delayed healing, crepitus in soft tissues, discolored or friable granulation tissue, and wound breakdown or dehiscence, or alternatively, the presence of fever and/or chills and at least one of these signs.^8,9^ Thereafter, in patients with local signs of infection, samples were obtained from local tissues, fluids, and blood and sent to the laboratory for bacterial culture and identification. In case of sterile microbiological cultures, the diagnosis of infection was assessed according to clinical and biological parameters. Indeed, initial antibiotic therapy can result in negative microbiological culture, and the prevalence of patients who developed wound infection secondary to snakebite could not be calculated as only those with positive microbiological cultures.^16,17^ Future studies should attempt to develop a more uniform set of criteria to define infection in snakebite envenomings to harmonize parameters that would allow comparison between studies.

**Table 4:** Bacteriological characteristics of wound infection following snakebite described in the literature

Reference	Chen et al.	Mao et al.	Hsieh et al.	Wagener et al.	Garg et al.	Sachett et al.	Jorge et al.	Our study
Year of publication	2011	2016	2017	2017	2009	2017	1994	2019
Geographic region	Taiwan	Taiwan	Taiwan	South Africa	India	Brazil	Brazil	Martinique
Responsible snake	*Trimeresurus mucrosquamatus*, *T. stejnegeri*, *Naja atra*	*Naja atra*	*Taiwan cobra*, *Bamboo viper*	*Naja mossambica*	–	*Bothrops* sp.	*Bothrops jararaca*	*Bothrops lanceolatus*
Number of bitten patients, *N*	231	112	148	164	–	187	–	170
Number of infected patients, *N* (%)	21 (9%)	86 (77%)	42 (28%)	42 (26%)	43	74 (40%)	40	20 (12%)
Number of positive samples, *N* (%)	21 (100%)	50 (58%)	21 (50%)	40 (95%)	43	6 (8.1%)	40	7 (35%)
Number of isolated strains, *N*	61	113	49	66	53	7	54	7
Aerobic Gram-positive bacteria, *N* (%)	14 (23%)	28 (24.8%)	13 (27%)	31 (47%)	28 (53%)	1 (14%)	11 (20%)	2 (10%)
* Enterococcus* species	12	21	11	31	4			
Coagulase-negative *Staphylococcus* species	1	4	–	–	5	–	–	–
* Bacillus* species	1	1	–	–	–	–	–	–
* Staphylococcus aureus*	–	2	2		17	1		
* Streptococcus* sp.	–	–	–	–	2	–	11	2
Aerobic Gram-negative bacteria, N (%)	39 (64%)	77 (68.1%)	24 (49%)	35 (53%)	25 (47%)	6 (86%)	37 (69%)	5 (25%)
*Acinetobacter baumanii*	–	1	2	–	2	–	–	–
* Aeromonas hydrophila*	–	7	1	–	–	–	–	3
* Citrobacter amalonaticus*	1	–	–	–	–	–	–	–
* Citrobacter freundii*	3	2	–	1	–	–	–	–
* Escherichia coli*	2	5	–	1	8	–	3	–
* Enterobacter* sp.	–	–	3	2	2	–	4	–
* Klebsiella pneumoniae*	1	1		1	4	–	–	–
* Morganella morganii*	14	32	12	17	3	6	23	2
* Proteus* spp.	5	8	3	10	3	–	–	–
* Providencia* sp.	3	6	–	–	–	–	7	–
* Pseudomonas aeruginosa*	5	6	–	–	3	–	–	–
* Salmonella enterica*	–	–	–	3	–	–	–	–
* Serratia liquefaciens*	1	1	–	–	–	–	–	–
* Serratia marcescens*	1	2	–	–	–	–	–	–
* Shewanella putrefaciens*	3	5	–	–	–	–	–	–
* Yokenella regensburgei*	–	1	–	–	–	–	–	–
Anaerobic bacteria, *N* (%)	8 (13%)	7 (6.2%)	3 (6%)	–	–	–	6 (11%)	–
* Bacteroides fragilis*	6	7	3	–	–	–	6	–
* Peptostreptococcus* sp.	2	–	–	–	–	–	–	–
Fungus, *N* (%)	–	1 (0.9%)	–	–	–	–	–	–
* Candida parapsilosis*	–	1	–	–	–	–	–	–
Others, *N* (%)	–	–	9 (18%)	–	–	–	–	–
No growth (% of infected patients), *N* (%)	0	36 (41.9%)	21 (50%)	2 (5%)	0	68 (91.9%)	0	13 (65%)

The main involved bacteria are *A. hydrophila* (Gram-negative bacilli), recognized to cause soft tissue infections and necrotizing fasciitis.^18^ *Aeromonas hydrophila* is generally found in sewage, freshwater, stagnant water, and feces. Other bacteria such as *M. morganii* have also been isolated in abscesses after *B. lanceolatus* bite. They are also found in the mouth and on the fangs of these viperids. Staphylococci, group D streptococci, *Clostridium*, *Escherichia coli*, and *Enterococcus faecalis*, involved in wound infection, have been also isolated from the mouth of viperid species.^19^ *Serratia marcescens* is rarely isolated from cellulitis following snakebite but may be responsible for bullous cellulitis. By contrast, *Staphylococcus aureus* is not commonly isolated from the snake mouth, suggesting that if the organism causes post-snakebite infections, it probably originates from the patient’s skin rather than having been inoculated by the snake fangs. Therefore, strict disinfection of the bite site should systematically be performed.^14^ The snake mouth is colonized by bacteria which can be transmitted to the bitten patient through the skin injury associated with the bite.^7^ Inoculation of bacteria from the mouth, fangs, or venom of *B. lanceolatus* following a bite can cause local infection with abscess and necrotizing fasciitis in the most severe cases, as described in other cases of snakebites.^20^ Based on the most frequently isolated bacteria in the snakebite site according to the literature (Table 4), active antibiotics include third-generation cephalosporins, piperacillin–tazobactam, and ciprofloxacin. Conformingly, in one recent study, isolated Enterobacteriaceae following snakebite infection showed 69% resistance to ampicillin, 60% resistance to amoxicillin–clavulanate, and 66% resistance to second-generation cephalosporins.^20^ By contrast, bacteria were sensitive to ceftriaxone in 97% of the cases and sensitive to ciprofloxacin and aminoglycosides in 100% of the cases. *Enterococcus faecalis* showed 92% sensitivity to ampicillin and amoxicillin–clavulanate and 100% sensitivity to ciprofloxacin. A recent experimental study examining the bacteria sampled from the oral cavity of 26 *B. lanceolatus* specimens collected from various areas in Martinique supported that microbiota from *B. lanceolatus* oral cavity was polymicrobial.^7^ The most frequently isolated bacteria were *A. hydrophila* (present in 50% of the samples), *M. morganii*, *K. pneumoniae*, *Bacillus* spp., and *Enterococcus* spp. Analysis of antibiotic susceptibility revealed that 67% of the isolated bacteria were resistant to amoxicillin–clavulanate. By contrast, most of the isolated bacteria were susceptible to third-generation cephalosporins (i.e., 73% to cefotaxime and 80% to ceftazidime). Similar data were also reported in the oral microbiota of snakes from Brazil and India.^19,21^

Despite snake oral and fang contamination with a wide variety of pathogenic bacteria, envenoming can be seen as a process associated with relatively limited risk of bacterial infection, except in cases associated with prominent tissue damage at the site of venom injection. Antibacterial effects of snake venoms may limit the likelihood of infection. Bactericidal activity against Gram-positive and Gram-negative bacteria was attributed to various components, including L-amino acid oxidases and phospholipase A2 enzymes.^22–27^ However, these bactericidal effects are likely to decrease once the venom has been injected. Soft tissue infection occurs in patients suffering severe envenomings (grade 3 or 4) in which the injected venom amount is likely to be high. Therefore, it is suggested that venom-induced skin and muscle damage is favorable for bacterial colonization and constitutes the bed of infection, as has been shown in an experimental model in mice.^28^

In the Practice Guidelines for the Diagnosis and Management of Skin and Soft Tissue Infections,^29^ use of antimicrobial agents active against both aerobic and anaerobic bacteria, such as amoxicillin–clavulanate, is recommended in bitten patients. However, the widespread systematic antibiotic administration is questionable after snakebite. Most authors recommend antibiotics in severely bitten patients, especially when local tissue damage occurs and inflammatory signs are suggestive of infection. Interestingly, empiric amoxicillin–clavulanate use was shown to be ineffective in preventing secondary infections from *Bothrops* snakebites because of the resistance to β-lactam antibiotics in the bacterial species commonly found infecting the snakebite site.^15^ Recently, analysis of the antibiotic susceptibility of bacteria isolated from *B. lanceolatus* mouth showed 67% of strains resistant to amoxicillin–clavulanate, whereas most isolated bacteria were susceptible to third-generation cephalosporins.^7^ In our hospital, empiric cephalosporin (or amoxicillin–clavulanate), aminoglycoside, and metronidazole combinations are routinely used in grade 3 or 4 envenoming and in case of clinical evidence of infection. Ciprofloxacin is the antibiotic of choice in case of allergy to β-lactams. This antibiotic treatment strategy probably explains the low prevalence of positive cultures (only 35%) from our patients’ samples in comparison to other reports in the literature (Table 4). However, we do not support the systematic antibiotic administration in all snake-bitten patients to reduce the risk of infection because such prophylactic use (including in non-severely envenomed patients) may have little impact on further infection but may give rise to side effects and select resistant organisms. Antibiotic administration should be considered only in patients having prominent local tissue damage and inflammation.

Our study has limitations. The diagnosis of wound infection involves repeated clinical assessment, biological dosing, and microbiological cultures. The involved bacteria were only identified in a limited number of cases having clinical evidence of infection possibly because of the difficulties of wound sampling in the emergency department and the fact that sample collection was performed after the antibiotic administration in some cases. This diagnostic approach is approved by many authors working on the diagnosis of wound infection and how to differentiate true infection from colonization.^8,9,16,17^ Further studies are needed to assess the sensitivity and specificity of clinical and biological parameters to assess the diagnosis of wound infection following snakebite independently of the microbiological results. In our study, anaerobic bacteria were not identified, although they are reported to be one of the responsible microorganisms causing cellulitis following snakebite. This is explained by the lack of bacteriological media for the isolation of anaerobic bacteria in our work. Our retrospective study methodology also limited further analysis. In addition, no clear indications and determined regimen of antibiotics were available, and treatment was only based on the judgment of the physicians in charge of the patients.

In conclusion, wound infection following *B. lanceolatus* bite is relatively frequent in grade 3 and 4 envenomed patients. The main involved bacteria are *A. hydrophila* and *M. morganii*. The empirical scheme for antibiotics adapted to the bacterial ecology of *B. lanceolatus* oral cavity are recommended for at least patients with grade 3 and 4 envenoming or having signs suggestive of local infection, regardless of the degree of envenoming. Our data support that the most appropriate empirical antibiotics are third-generation cephalosporins and that empirical amoxicillin–clavulanate should no longer be used in this context.
